# 2225. Implementing Updated Intraamniotic Infection Guidelines at a Large Academic Medical Center

**DOI:** 10.1093/ofid/ofad500.1847

**Published:** 2023-11-27

**Authors:** Casey Smiley, Jessica Rizzuto, Nicola C White, Christina T Fiske, Jennifer Thompson, Benjamin Ereshefsky, Milner Staub

**Affiliations:** Vanderbilt University Medical Center, Nashville, Tennessee; Vanderbilt University Medical Center, Nashville, Tennessee; Vanderbilt University Medical Center, Nashville, Tennessee; Vanderbilt University Medical Center, Nashville, Tennessee; Vanderbilt University Medical Center, Nashville, Tennessee; Vanderbilt University Medical Center, Nashville, Tennessee; Vanderbilt University Medical Center, VA Tennessee Valley Healthcare System, Nashville, TN

## Abstract

**Background:**

Intraamniotic infection (IAI) affects 2-5% of laboring patients and has significant neonatal and maternal morbidity. The American College of Obstetricians and Gynecologists (ACOG) suggests ampicillin and gentamicin as treatment but allows consideration of local antibiotic resistance in antibiotic choice. Given toxicity and rising resistance to gentamicin and ampicillin, we aimed to update local guidelines for IAI treatment to piperacillin-tazobactam.

**Figure 2:**
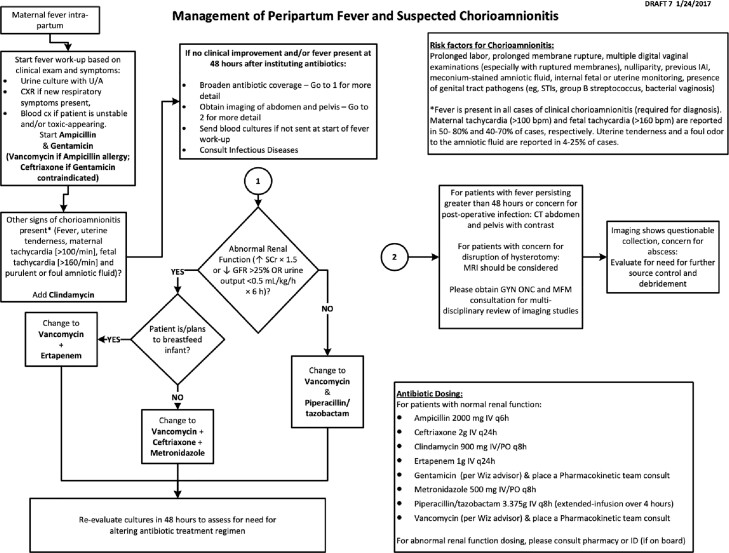
Pre-existing Intraamniotic Infection Protocol

The prior internal protocol in place for suspected intraamniotic infection, consistent with ACOG primary recommendations.

**Figure 3:**
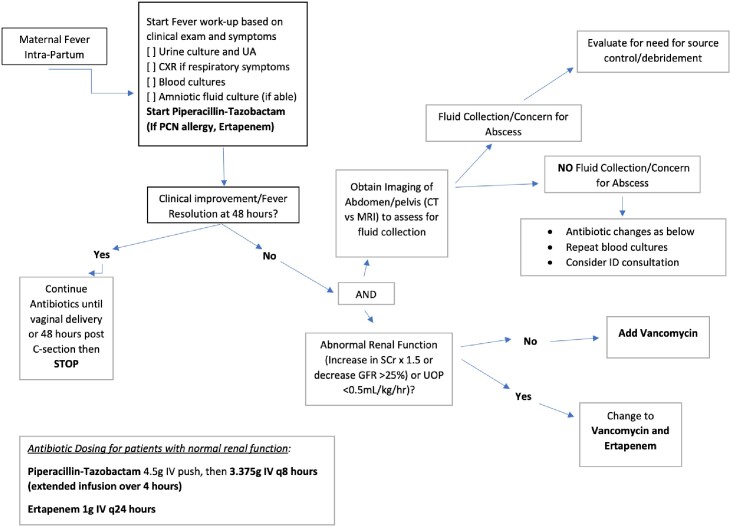
Updated Intraamniotic Infection Protocol

Our updated protocol for intraamniotic infection, based off of the local antibiogram.

**Methods:**

Institutional antibiogram data and ACOG recommendations informed guideline changes to improve bacterial coverage and reduce exposure to nephrotoxic/ototoxic agents. A pre-implementation survey assessed provider satisfaction and knowledge of current guidelines. Using the inner Behavior Wheel Change framework for understanding behavior, we identified barriers to changing prescribing behavior. Interventions were designed and bundled using the hierarchy of intervention effectiveness (Fig 1). A clinical algorithm was developed and implemented into an existing electronic order set (Fig 2,3). Antibiotic use in days of therapy (DOT) per 1000 patient days present (PDP) pre- and post-study implementation were collected and measured.

**Figure 1:**
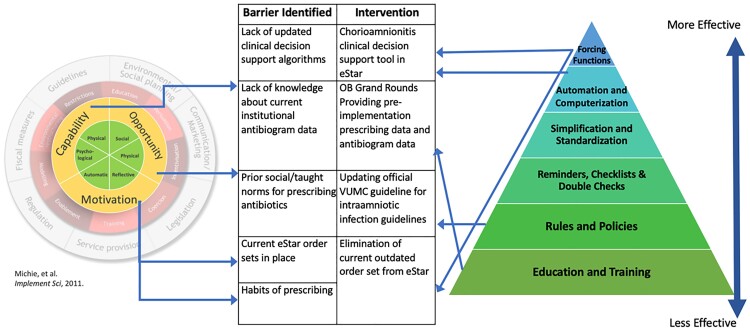
Quality Improvement Barriers and Interventions

The inner behavior wheel of change was used to identify barriers to implementation. Interventions were assessed and designed using the hierarchy of intervention effectiveness.

**Results:**

The pre-implementation survey had a 49% (61/142) response rate with 75% feeling confident ordering antibiotics for IAI ≥ 75% of the time. However, 52% reported feeling satisfied ≤ 50% of the time with the current antibiotic ordering process. Only 49.2% were aware of internal IAI guidelines.

Following implementation, gentamicin use decreased from 15.0 DOT/1000 PDP in the 6 months prior to implementation, to 1.0 DOT/1000 PDP in the 3 months post-implementation. Piperacillin-tazobactam use increased from 14.3 DOT/1000 PDP pre-implementation to 33.6 DOT/1000 PDP post-implementation (Fig 4).

**Figure 4:**
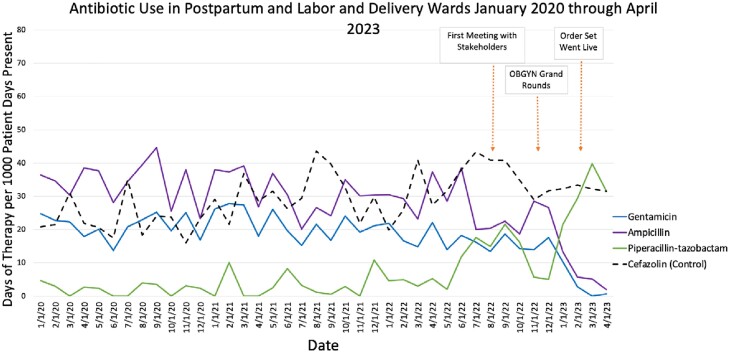
Antibiotic Use in Postpartum and Labor and Delivery Wards January 2020 through April 2023

Days of therapy (DOT) per 1000 patient days present (PDP) for aminoglycosides, ampicillin, and piperacillin-tazobactam in postpartum and labor and delivery wards 1/1/2020 through 4/2023. Cefazolin is shown as a control, as this was not a target of intervention, and remains as a standard pre-operative antibiotic.

**Conclusion:**

Clinical decision support tools, an example of forced function interventions, were used for successful implementation of updated antibiotic guidelines for IAI, facilitated by collaboration with stakeholders and widespread education. Further analysis of their long-term impact on antibiotic use and any potential for unintended patient harm are planned. Impact on stakeholders is being assessed via a post-implementation survey.

**Disclosures:**

**Jennifer Thompson, MD**, Up To Date Contributor: Honoraria **Milner Staub, MD, MPH**, Gilead: Stocks/Bonds|Johnson & Johnson: Stocks/Bonds

